# “Emerging role of plasma kallikrein inhibitors in preventing hereditary angioedema flares in pregnancy”

**DOI:** 10.1097/MS9.0000000000004663

**Published:** 2026-01-14

**Authors:** Nazeer Mustafa, Rabia Ramzan, Javed Iqbal, Hamza Aka Khail

**Affiliations:** a Dow Medical College, DUHS, Karachi, Pakistan; b King Edward Medical University, Lahore, Pakistan; c Department of Physiotherapy, DUHS, Karachi, Pakistan; d Department of Medicine, Kateb University, Afghanistan

**Keywords:** fetal safety, hereditary angioedema, long-acting prophylaxis, Navenibart, plasma kallikrein inhibitor

## Abstract

Hereditary angioedema (HAE) presents a significant therapeutic challenge during pregnancy, a period often associated with increased attack frequency and severity. The management of HAE in pregnancy is complicated by physiological changes and limited fetal safety data for many prophylactic agents. Recent advancements in long-acting prophylaxis, particularly monoclonal antibodies targeting plasma kallikrein such as Navenibart (STAR-0215), offer a promising new therapeutic approach. With an extended half-life of up to 105 days, Navenibart enables infrequent dosing (every 3–6 months), potentially reducing treatment burden and improving coverage through critical gestational periods. However, its use in pregnant patients remains strictly investigational, as no clinical safety or efficacy data exist for this population. This article examines the emerging role of plasma kallikrein inhibitors in HAE prophylaxis during pregnancy, discusses the pharmacologic profile and potential advantages of Navenibart, and proposes cautious, preliminary clinical considerations within a shared decision-making framework. Multidisciplinary care and further research are urgently needed to establish safety and define the place of these agents in obstetric HAE management.

*Dear Editor,*

Hereditary angioedema (HAE) is a rare, autosomal-dominant illness characterized by recurrent, unpredictable episodes of swelling that can be fatal, particularly when affecting the upper airway^[^1,2^]^. HAE poses a substantial therapeutic challenge during pregnancy^[^3^]^. Large retrospective studies consistently show that 59–67% of pregnancies in HAE patients are associated with an increased frequency of attacks^[^4,5^]^. Furthermore, attack severity often worsens during pregnancy and breastfeeding compared to pre-pregnancy periods^[^5^]^. The management of HAE in pregnant patients is therefore a complex clinical challenge, and its therapeutic arsenal is restricted by limited safety data for the fetus^[^3^]^.

HAE significantly impacts both maternal and fetal health. Uncontrolled swelling attacks can lead to severe discomfort, dehydration due to gastrointestinal issues, and pose risks of laryngeal edema and asphyxiation^[^1,2^]^. For the fetus, maternal hypoxemia during a severe attack poses a direct threat. Furthermore, the stress of repeated attacks may contribute to an increased risk of adverse pregnancy outcomes. Many traditional long-term prophylactic agents have limitations in this context. Attenuated androgens are contraindicated due to their virilization potential on the fetus, while the safety data for tranexamic acid remain insufficient^[^6^]^. Although plasma-derived C1-inhibitor (pdC1INH) is often considered the cornerstone of prophylaxis during pregnancy^[^4,6^]^, its short half-life necessitates frequent intravenous or subcutaneous administrations (every 3–4 days), creating a substantial treatment burden^[^7^]^.

To ensure clarity, transparency, and repeatability in the usage and reporting of AI-based approaches, this work was published in compliance with the Transparency in the Reporting of Artificial Intelligence (TITAN) guideline^[^8^]^.

## Navenibart-plasma kallikrein inhibitor (STAR-0215)

The recent development of long-acting prophylactic agents, specifically monoclonal antibodies targeting plasma kallikrein, offers a novel therapeutic approach with the potential to address this unmet need. The phase 1a first-in-human study of Navenibart (NCT05695248) reported a remarkably extended mean half-life of 82–105 days following a single subcutaneous dose (≥300 mg) and provided durable, statistically significant inhibition of plasma kallikrein activity for up to 224 days^[^9^]^. This pharmacokinetic (PK) and pharmacodynamic profile is unprecedented among HAE therapies. Crucially, PK modeling from this study supports the feasibility of every-3-month (Q3M) or even every-6-month (Q6M) maintenance dosing regimens after a loading dose^[^9^]^.

This infrequent dosing schedule has profound implications for pregnancy management. For a patient planning a pregnancy, initiating a long-acting prophylactic like Navenibart could provide continuous protection throughout the critical first trimester and beyond with a single injection, eliminating the treatment gaps and logistical hurdles associated with frequent dosing. This is a significant advantage over current options like Lanadelumab, which requires subcutaneous injection every 2–4 weeks, or Berotralstat, a daily oral medication whose use in pregnancy is not yet established^[^10^]^.

Furthermore, the mechanism of action of Navenibart, a highly specific, allosteric inhibition of plasma kallikrein, and its design with an Fc YTE modification to extend half-life, suggests a targeted therapeutic approach with a potentially favorable safety profile^[^11^]^. The phase 1a data reported a safety profile consistent with other injectable HAE therapies, with no serious adverse events and mostly mild, transient injection site reactions^[^9^]^.

However, it is crucial to emphasize that these potential benefits remain theoretical. The cited phase 1a study was conducted in healthy adults, and no clinical data exist regarding the safety, efficacy, or PK of Navenibart in pregnant women. While the monoclonal antibody platform is used in other chronic conditions during pregnancy (e.g., inflammatory bowel disease and rheumatological conditions), the specific safety profile of Navenibart for the fetus is unknown. The potential for placental transfer, particularly in the third trimester, and any long-term developmental effects require rigorous investigation. Therefore, while the mechanism of action (highly specific plasma kallikrein inhibition) and extended dosing interval make Navenibart a compelling candidate, its application in obstetric care must be considered strictly investigational at this time.

## Guidelines recommendation

Based on this emerging evidence and the well-documented challenges of HAE in pregnancy, we propose preliminary and cautious guidelines for the consideration of long-acting plasma kallikrein inhibitors in obstetric care. These recommendations are speculative and should be applied only within a shared decision-making framework, acknowledging the current lack of comprehensive pregnancy safety data.

1. **Preconception Counseling and Planning: A Cautious Approach.** Women with HAE of childbearing potential should be counseled about all prophylactic options, including the investigational status of long-acting agents like Navenibart. For patients planning a pregnancy who are at very high risk for severe attacks, and for whom standard therapies are ineffective or intolerable, initiating a long-acting prophylactic may be considered before conception to establish stable disease control. This decision requires extensive discussion of potential unknown risks versus benefits^[^5,12^]^.
2. **First-Trimester Management: Continuation with Explicit Justification.** A patient who presents pregnant while already on a stable regimen of a long-acting plasma kallikrein inhibitor should be managed individually. Discontinuation should be considered based on attack history and gestational age. If continuation is deemed necessary to prevent life-threatening maternal attacks, this must be explicitly justified in the medical record. The potential danger of minimal fetal drug exposure from a long-half-life agent must be weighed carefully against the documented high risk of severe maternal morbidity from uncontrolled HAE.
3. **Dosing Strategy: Minimizing Exposure as a Primary Goal.** The prolonged dosing interval should be used strategically with the explicit goal of minimizing the total number of injections during pregnancy. Close coordination with the obstetric team is mandatory to time doses to avoid, if possible, the peripartum period when a dose is due. This aims to reduce confounding variables during delivery and the immediate postpartum phase.
4. **Multidisciplinary Care and Vigilance.** The evaluation of these agents in pregnancy demands a collaborative model involving allergist/immunologists, maternal-fetal medicine specialists, pharmacologists, and obstetric anesthesiologists to design appropriate studies and manage complex cases. A well-defined, documented strategy for handling breakthrough attacks and the postpartum phase is essential. Enhanced fetal monitoring may be prudent given the lack of long-term safety data.

## Conclusion

HAE poses significant therapeutic challenges during pregnancy, marked by increased attack frequency and severity. Management is complicated by physiological changes and limited fetal safety data for prophylactic agents. Recent advancements include long-acting monoclonal antibodies targeting plasma kallikrein, such as Navenibart (STAR-0215), which allows infrequent dosing and may ease treatment burdens. However, its use in pregnant patients lacks clinical safety or efficacy data and remains investigational. This article explores the role of plasma kallikrein inhibitors in HAE prophylaxis during pregnancy, discussing Navenibart pharmacological profile, potential advantages, and recommending cautious clinical considerations. Multidisciplinary care and further research are crucial for establishing safety and integrating these agents into obstetric HAE management.

## Data Availability

Not applicable.

## Gurantor

Nazeer Mustafa.

## Clinical trials

Trial Registry. Astria Therapeutics, Inc (NCT05695248) (STAR-0215).
